# Assays for Determining Pertussis Toxin Activity in Acellular Pertussis Vaccines

**DOI:** 10.3390/toxins11070417

**Published:** 2019-07-17

**Authors:** Kevin Markey, Catpagavalli Asokanathan, Ian Feavers

**Affiliations:** Division of Bacteriology, National Institute for Biological Standards and Control (NIBSC), Blanche Lane, South Mimms, Potters Bar, Hertfordshire EN6 3QG, UK

**Keywords:** pertussis toxin, acellular pertussis vaccine, vaccine safety, histamine sensitization test, CHO cell clustering assay, in vitro alternative methods

## Abstract

Whooping cough is caused by the bacterium *Bordetella pertussis*. There are currently two types of vaccines that can prevent the disease; whole cell vaccines (WCV) and acellular vaccines (ACV). The main virulence factor produced by the organism is pertussis toxin (PTx). This toxin is responsible for many physiological effects on the host, but it is also immunogenic and in its detoxified form is the main component of all ACVs. In producing toxoid for vaccines, it is vital to achieve a balance between sufficiently detoxifying PTx to render it safe while maintaining enough molecular structure that it retains its protective immunogenicity. To ensure that the first part of this balancing act has been successfully achieved, assays are required to accurately measure residual PTx activity in ACV products accurately. Quality control assays are also required to ensure that the detoxification procedures are robust and stable. This manuscript reviews the methods that have been used to achieve this aim, or may have the potential to replace them, and highlights their continuing requirement as vaccines that induce a longer lasting immunity are developed to prevent the re-occurrence of outbreaks that have been observed recently.

## 1. Introduction

### 1.1. Pertussis Disease

The Gram-negative bacterium *Bordetella pertussis* is the aetiological agent of whooping cough. This is a serious, highly contagious respiratory disease, especially dangerous in infants who are either too young to have been vaccinated or have not received their full vaccination series [1]. The disease normally presents as a paroxysmal cough with a characteristic whoop. The cough can be followed by vomiting and symptoms can last for several months. More severe complications include pneumonia, pulmonary hypertension, febrile seizures, encephalopathy and brain haemorrhages [2,3]. There are several virulence factors associated with *B. pertussis* such as pertussis toxin (PTx), dermonecrotic toxin, tracheal cytotoxin and adenylate cyclase toxin. Proteins that are involved in cell binding such as filamentous haemagglutinin (FHA), fimbriae (FIM) and pertactin (Prn) also play a role in pathogenesis [3,4,5]. The production of these factors is controlled by a two-component signal transduction system (BvgAS) which is regulated by environmental factors in the host such as temperature [6,7,8]. The initial step in pathogenesis is adherence to tracheal epithelium followed by colonisation leading to local damage in the airways. The age and immunocompetence of the patient have an effect on the severity of disease with infants most seriously affected [6].

### 1.2. Pertussis Vaccines

The first vaccines developed to prevent pertussis infection consisted of killed whole cells (WCV). These have been widely administered internationally since the 1940s, and their use has resulted in a marked reduction in the morbidity and mortality associated with pertussis disease [9,10]. They are still the most commonly administered type of pertussis vaccine globally mainly due to their lower cost [11,12]. Whole cell vaccines comprise specific strains that are grown in conditions that ensure the expression of the virulent phase 1 phenotype. The strains are then subsequently heat- or formalin inactivated [4,13]. The strains used are chosen to include the agglutinogen serotypes 1, 2 and 3. While one strain may cover all three types, usually a combination of different strains expressing serotypes 2 and 3 are used in the production of WCV as all *B. pertussis* strains express agglutinogen 1. Whole cell vaccines induce mainly cellular based immune responses resulting in long lasting immunity in humans and have been demonstrated to prevent carriage in a non-human primate model [5,14].

However, some side-effects have been associated with the administration of WCVs. These include mild symptoms such as, redness around the injection site to very rare severe symptoms such as febrile seizure [15,16,17,18]. Although these reactions had no long term sequalae they resulted in the suspension of WCV programs in some countries and had a negative effect on vaccine compliance in others [19,20,21,22]. This led to the development of two types of acellular pertussis vaccines (ACV) that are less reactogenic. The first type was developed in Japan as co-purified preparations of PTx and FHA [21,23]. The second type contains more highly purified PTx and FHA along with other *B. pertussis* antigens in many different concentrations and combinations [24,25]. The main component present in all ACV vaccines is PTx in its detoxified form (PTd). Most ACV products also contain other purified pertussis antigens such as FHA, Fim 2&3 and Prn as these antigens are considered to be important in the generation of a protective immune response [26]. The purification process means that reactogenic components of the WCV vaccine, such as lipopolysaccharide, are omitted from the final vaccine formulation. The resulting ACVs are far less reactogenic with no serious side effects, while remaining immunogenic [26]. For the final vaccine formulation pertussis antigens are adsorbed to an adjuvant, which are normally alum based such as aluminium phosphate or aluminium hydroxide. Finally, ACVs are also usually formulated in combination with components to protect against other diseases including tetanus and diphtheria toxoids, inactivated polio virus and *Haemophilus influenza B* polysaccharide [27,28,29].

Since the widespread introduction of vaccination against pertussis there has been a marked reduction in the number of cases in whooping cough, but recent years have seen outbreaks in areas with a high ACV vaccination coverage such as Europe, USA and Australia [30,31,32,33,34,35,36,37,38]. There are number of possible reasons for these outbreaks and increased number of cases. Firstly, ACVs predominantly induce a humoral, or Type 2 T helper cell (Th2), immune response as opposed to the cellular Th1 and Th17 immunity induced by WCV and natural infection and which are associated with protection and clearance. This Th2 response may also result in poorer long-term protection compared to WCV [5,6,39,40,41,42,43]. Furthermore, it has been demonstrated in baboon models that ACVs do not prevent colonisation and transmission [14]. Also, bacterial isolates that do not express the antigens used in ACVs, such as Prn and even PTx, have been identified in a number of countries along with antigenic variation may result in vaccine escape [41,44,45,46,47,48,49]. Finally, improved awareness and surveillance methods may also have resulted in the identification of cases that would otherwise have been undiagnosed previously [50,51]. For these reasons, there is continued interest in developing improved ACVs, less reactogenic WCVs, and outer membrane vesicles-based vaccines among others [3,13,52,53].

### 1.3. Structure of PTx

PTx is a multimeric 105 kDa protein and has an A-B type structure (AB_5_) typical of many other bacterial toxins, including cholera toxin [54]. The A-protomer is a monomer and is enzymatically active. The B-oligomer binds to carbohydrates on the target cell membrane. While the A-protomer of PTx is comprised of just one subunit peptide (S1) the B-protomer has five subunits of S-2 through to S-5 with two S-4 subunits [55,56,57]. Intact B-oligomer is required for binding of the toxin to the cell surface and enables entry of the A-protomer into the cells by translocation across the plasma membrane by retrograde transport [58,59]. This process involves the A-subunit moving through the pore which is created in middle of the B-pentemer [60]. Once inside the cell, the A-subunit is trafficked to the Golgi apparatus and then on to the endoplasmic reticulum (ER) where the A-protomer dissociates from the rest of the toxin. The A-subunit is then exported to the cytoplasm through ER-associated degradation (ERAD) [61,62]. In the cytoplasm the enzymatic activity of the A-subunit ADP-ribosylates G-proteins [63,64,65]. This results in a wide range of physiological effects including lymphocyctosis, insulin secretion, histamine sensitization, mitogenesis, melanocyte stimulation, T and B-lymphocyte stimulation, inhibition of neutrophil chemotaxis and monocyte migration [59,66]. Even though it has been demonstrated that the binding of the B-subunit only has a physiological effect for monitoring PTx activity in the context of vaccine safety both A and B subunit functionality need to be assessed.

In its native form PTx is too toxic to be used directly in vaccines and therefore must be carefully detoxified whilst ensuring that the molecule retains its protective immunogenicity. The detoxification process therefore needs to be well controlled to result in a stable product that is sufficiently detoxified and does not revert back to toxicity while maintaining its protective epitopes. Detoxification is primarily a chemical process. Most commonly glutaraldehyde and formaldehyde, either individually or in combination, are used in detoxification. The process of chemical detoxification typically involves incubating the toxin with glutaraldehyde for 2 h at 25 °C or formaldehyde at 40 °C for 7 days [67]. Detoxification by hydrogen peroxide is also used, for example in Denmark, and has been shown to result in less disruption of epitopes compared with formaldehyde treatment [68,69,70].

Chemical detoxification usually results in the crosslinking of amino acid residues of the toxin but different procedures using different reagents and conditions have been shown to result in distinct conformational changes and epitope binding patterns [71,72,73,74,75,76,77]. Glutaraldehyde treatment results in the crosslinking of lysine residues. There are no such residues in the S1 subunit of PTx but several in the subunits that comprise the B-oligomer [78,79]. Glutaraldehyde detoxification results in the loss of the molecule’s ability to bind to the cell membrane and hence enter the cell to express its toxic activity. The ADP-ribosyl transferase activity is not affected by glutaraldehyde detoxification [67].

Formaldehyde reacts with the amino group of the N-terminal and the side-chains of arginine, cysteine, histidine, and lysine residues [75]. Since most of these residues are found in both A and B subunits treatment with formaldehyde leads to a reduction of both binding and enzymatic activities of the toxin [67]. Formaldehyde treatment also results in the loss of ability of the protective monoclonal antibody 1B7 to bind to the toxin [74,80].

A number of genetically modified PTxs (PTgs) have also been developed whereby mutations in the A subunit have resulted in a loss of toxicity. These toxoids have two amino acid substitutions in the active site of the enzyme that result in the loss of ADP-ribosylation activity. They have been found to be safe and elicit an immunogenic response similar to, or better than, the native PTx antigen [81,82,83,84,85,86,87]. It may also be possible to engineer *B. pertussis* strains that express PTg in higher amounts than native strains [88,89].

Given the delicate balance that needs to be achieved to reduce toxicity to a level proven to be safe in clinical trials while retaining protective immunogenicity, the ability to accurately measure residual PTx activity is critical to ensure ACV vaccines are safe. It is also important to demonstrate the stability of the detoxification process thus ensuring that the toxoid does not revert back to toxicity during storage. To confirm stability and the absence of reversion final ACVs are often incubated at 37 °C for 4 weeks and the PTx activity assessed [90,91,92]. Therefore, methods to accurately measure any PTx activity that may be present in bulk toxin and toxoid preparations, and in final formulations of vaccines are required to ensure vaccine safety and monitor batch-to-batch variation [4].

## 2. Specific Tests for PTx Toxicity

### 2.1. Lethal Histamine Sensitization Test (Lethal HIST)

Following the introduction and widespread use of ACVs the lethal histamine sensitization test (HIST) in mice was for a long time the only means for detecting residual PTx toxicity in final products along with in toxin and toxoid bulks. It was first developed to detect PTx activity in WCV vaccines but was subsequently used for ACV products in Japan, then the US and Europe [93]. It is based on the principle that mice are inherently resistant to histamine toxicity but become sensitive after exposure to PTx [93,94,95,96,97].

There are many variations of the lethal HIST used globally (Table 1), however, they broadly follow a similar format. Mice are divided into groups of pre-defined numbers which receive either a dose of test vaccine stored at 2–8 °C to check for residual toxicity or vaccine incubated at 37 °C for 4 weeks to monitor reversion to toxicity. Groups may also receive a reference vaccine with established clinical safety for comparison. All forms of the assay also include groups which receive active PTx as positive control at a dose that has been defined to cause death in pre-defined number of mice to ensure the sensitivity of the mice to histamine challenge. Three to five days later the mice are challenged with histamine and monitored for 24 h. If the mice have been exposed to active PTx they can experience anaphylactic shock leading to a reduction in body temperature, pain, distress and death. The number of deaths in each group following challenge are recorded. The validity criteria of the various form of the assay also vary as does the acceptance criteria for the test vaccines to pass (Table 1). Generally, deaths are required in the positive control groups to demonstrate that the mice are sensitive to the histamine challenge and the level of PTx activity (i.e., deaths) in the test vaccine groups should be at, or below, an acceptable level or reference vaccine preparation [91,92,98].

### 2.2. Temperature Histamine Sensitization Test (Temperature HIST)

A refinement to the lethal HIST assay was developed and used successfully in Japan and other countries in Asia for testing ACVs. This assay is based on the reduction in body temperature observed in PTx exposed mice following challenge with histamine. It uses similar groups and procedures as the lethal HIST but the body temperature of the mice is measured 30 min after histamine challenge [99]. Temperature can be measured either with a rectal/dermal thermometer or a thermal camera. This method provides a quantitative estimate of the PTx activity of a test vaccine relative to a PTx reference standard which has been assigned histamine sensitization units (HSU) or International Units (IU). The accepted level of PTx in test vaccines also varies by region with 0.4 HSU the level in Japan and 0.8 HSU in China (Table 1) [90,100,101,102]. The animals can then be culled once the temperature reading has taken place and thus reduce the severity of the procedure. It is, however, still considered a severe assay from an animal welfare viewpoint as a large proportion of the mice that die in the assay do so in the first 30 min after challenge [99].

Both forms of the HIST assay can be used to measure the levels of PTx activity in samples by comparing the drop in temperature, or deaths per group, for a range of doses to the responses in a reference preparation [103,104,105]. While both death and changes in body temperature have been found to be dose dependent they do not seem to be linked and the modes of action may not be the same [99,102].

There are a number of advantages associated with the HIST assays. Firstly, HIST captures all activities of the toxin; binding to the cell membrane, translocation and enzymatic activity once intracellular. The mode of action of PTx in the lethal HIST is thought to mirror that in humans in that the primary mechanism is cell intoxication and the ADP-ribosylation of G proteins in vascular smooth muscles leading to vasodilation and hypovoleamic shock that causes death [93,107,108,109]. Secondly it is very sensitive for detecting PTx activity. Thirdly, other vaccine components, such as alum adjuvants, do not interfere with the mode of activity. Finally, the potential toxin bioavailability of the vaccine in the mice is a reasonable mimic of the environment of the human vaccine recipient [110].

It has been estimated that the limit of quantification (LOQ) of the HIST assay varies for different PTx materials. For the WHO 1st International Standard for PTx (JNIH-5) the LOQ was estimated to be 1.22 IU per animal and 1.11 IU per animal for European Directorate for the Quality of Medicines and HealthCare’s (EDQM) BRP batch 1 [111]. These estimates were calculated using data published in an EDQM collaborative comprising 25 assays and three mouse strains [105,111]. The EDQM study also estimated that the highest level of residual PTx activity in the licenced ACV based combination vaccines included in the study is approximately 2 IU/SHD [105].

There are several disadvantages associated with the HIST assays. Firstly, while the mechanism of the lethal assay is better understood than the temperature method the mechanisms of both forms are poorly understood. It is also believed that the modes of action of the two methods are not linked as there is no temperature drop that is predictive of death [93]. Secondly, they have poor reproducibility leading to high intra- and inter-laboratory variability. This variability can result in significant repeat testing resulting in the use of many mice. There are many factors that result in a high degree of variation of the assay between laboratories for both forms of HIST assay. These include differences in species, age and gender of the mice used, the time between administering the test samples and the histamine challenge, the dose of histamine and control groups included. A number of international collaborative studies, including the recent study for the establishment of the 2nd WHO International Standard for PTx, have highlighted the large differences in HIST assays performed by laboratories globally [103,104,105]. Also, Table 1 gives an indication of the various forms if HIST used globally. Also, the fact that a single batch going to multiple markets must pass the various versions of HIST relevant to each market substantially increases the number of animals used. It has recently been estimated that approximately 65,000 mice are used globally in HIST assays per year [102]. On top of this the large number of variations in HIST make it difficult to standardize [4]. Finally, there are ethical concerns about animal welfare as the test can cause severe pain and distress, especially for mice receiving a PTx reference standard. For these reasons HIST has long been regarded as a priority for replacement with in vitro alternative assays [4,67,93,102,110,111,112]. However, following consultations between vaccine manufacturers and national control laboratories, the requirement for testing for residual and reversion to toxicity in final formulated vaccines will be removed from the European Pharmacopoeia in 2020 once the detoxification process has been demonstrated to be robust and stable [106]. Tests for residual toxicity and reversion to toxicity will still be required for new products.

### 2.3. CHO Cell Clustering Assay

Chinese Hamster Ovary (CHO) cells have been used as a bioassay system for other AB_5_ toxins such as cholera toxin. In the presence of cholera toxin CHO cells become stretched and elongated [113,114]. Hewlett et al. first described a test for PTx activity using CHO cells. They observed that in the presence of active PTx the cells also exhibited a change in morphology but instead became rounded and formed clusters [115]. This altered morphology was found to be due to the ADP-ribosylation activity of PTx on the cells. It was also found that A-subunit and B-subunit individually exhibited greatly reduced clustering activity compared to the whole molecule. Toxoid forms of PTx also had reduced activity [116]. The assay was developed in a microtitre plate format and has been used to monitor the PTx content of upstream vaccine components such as PTx and PTd non-adjuvanted bulks [91,98,117]. In the test a monolayer of CHO cells is treated with vaccine or reference PTx at a range of dilutions. After incubation the plates are observed using an inverted microscope and scored for clustering. The highest dilution of the test sample that results in clustering represents the titre. In this way the test can provide a semi-quantitative estimate of the PTx content of the sample when analysed alongside a reference purified native PTx preparation. Initially the variability of the CHO assay was found to be greater than the HIST in a number of collaborative studies to calibrate different reference preparations of PTx [104,105]. However, like HIST, this was most likely due to the large differences in reagents and methods used by different participants despite early efforts to develop optimised standard assay conditions [117,118,119]. To address this high variability a standardised method was developed by the EDQM under the aegis of the Biological Standardisation Programme (BSP). In this standardised method CHO-K1 cells should be obtained from specific sources, they should be propagated in a defined medium using a standardised number of passages and the scoring of cells for clustering should follow a defined procedure. This standardised method gave good inter-laboratory reproducibility and transferability for pure PTx [120]. It and other CHO cell clustering assay protocols were not, however, suitable for testing residual PTx activity in final vaccine formulations because some components of the vaccines, such as alum-based adjuvants, proved to be cytotoxic to the CHO cells [105,112,120]. Recently, two modified versions of the assay have been developed that attempt to allow testing of final formulations by overcoming these cytotoxic effects [120]. These methods are referred to as the Direct and Indirect methods and were also developed under the auspices of the EDQM. For the Direct method vaccine preparations are sufficiently diluted to negate the toxic effects of vaccine components and the diluted samples are added directly to the cells. With the Indirect method the vaccine preparations are centrifuged, and the supernatant discarded before the pellet is resuspended in culture media. The resuspended pellet is then added to microtitre plate wells which contain a semi-permeable membrane. This membrane is above the CHO cells and prevents the vaccine adjuvant coming into contact with them [120]. A comparison of the modified CHO assays was performed in an EDQM study (BSP114) using a common set vaccine samples spiked with known concentrations of PTx. Most of the participants who performed the Direct method observed cytotoxic effects in wells with diluted vaccine. All laboratories that performed the Indirect method produced interpretable results and while there was some variation, the majority of assays were able to identify vaccines spiked with 1–4 IU/mL [112,120].

One of the main advantages associated with the CHO cell clustering assay is that it captures the cell binding, translocation and enzymatic activity of the toxin and therefore is comparable to HIST [110,116]. It is also very sensitive and detects lower levels of pure PTx than HIST [121]. However, there are several disadvantages associated with the different forms of CHO cell clustering assays. Firstly, it is a subjective decision by the operator to identify when cells are exhibiting a clustering morphology and hence determine the cut-off titre which is the final readout of the assay. This subjectivity results in a great deal of variability [122]. It is for this reason, two operators are required in the standardised methods to read the plates and determine the final titres at which clustering is observed and recorded [103,123]. There is also concern with the Indirect method that any residual PTx activity in the supernatant is not measured in this assay however it could be monitored using alternatives in vitro assays such as carbohydrate binding or ADP-ribosylation [112]. Also, there have been reports that the CHO cell clustering assay failed detect reversion to toxicity of a batch of ACV that failed in the HIST assay. It also failed to detect activity in toxoids that demonstrated some toxicity in the HIST assay [124].

Reference preparations of active PTx with assigned potency values are required as controls in both HIST and CHO assays. They are also important for inter-assay and inter-laboratory comparability. Recently, the standardised CHO cell clustering method was used in the calibration of the current WHO International Standard for PTx (NIBSC code 15/126) and to calibrate EDQM’s Biological Reference Preparation (BRP) batch 1 in a joint study [103,123]. This study found that the biological activity measured by the CHO cell assay does not always correspond to the activity found in HIST when different PTx reference preparations are compared. When the first WHO International Standard (IS) for PTx (JNIH-5) was established it was found that the activities in the lethal HIST and CHO assays relative to other PTx preparations were approximately the same and therefore the standard was assigned a unitage of 10,000 IU/ampoule for both assays [104,121]. However, when the replacement for JNIH-5 was being established it was found that the activity of the replacement, 15/126, was approximately one fifth of JNIH-5 in the lethal HIST assay but 1/15 the activity of JNIH-5 in the CHO cell assay [103]. A similar discrepancy was observed to BRP batch 1 in both assays [105,123]. The discrepancy between the HIST and CHO assays is probably not unexpected as the activity of biological substances depends on their interaction with the test systems and in the case of two different materials the tests may favour the activity of either one or other of the material [125].

### 2.4. Biochemical Assays

Cyr et al. (2001) first developed an assay to measure the enzymatic activity of the A subunit of PTx [126]. In this assay the ADP-ribosylation activity is measured using a synthetic homologue of a G-protein which is tagged with a fluorescent marker. In the presence of nicotinamide adenine dinucleotide (NAD) the A-subunit of PTx transfers the ADP-ribose of the NAD to the cysteine moiety of the synthetic protein. This ADP-ribosylated product can then be separated from the substrate using reverse-phase HPLC and a fluorescent detector. It has been found that the response is linear for native pure PTx at concentrations ranging from 0.0625 to 4.0 µg/mL [126]. The concentrations of some components of the assay were then slightly modified to assist with the successful method transfer between laboratories [127]. It was then used in an international collaborative study for the establishment of an EDQM PTx BRP [128]. It was during this, and subsequent studies, that it was observed that the ADP-ribosylation activity did not always correlate with residual PTx activity found by HIST in vaccine products [128,129]. It was also found that different ACV products had different amounts of ADP-ribosylation activity and a level that would be significant in comparison to the HIST could not be defined. Different detoxification methods as well as different final vaccine formulations may result in this lack of correlation between ADP-ribosylation and HIST. Therefore, it was clear that relying solely on ADP-ribosylation activity may not fully reflect what is observed in the HIST for chemically detoxified products as cell binding and internalisation are required for subsequent ADP-ribosylation to occur [129]. To overcome this limitation a test was developed which measures the activity of the B-subunit [130].

Since the B-oligomer of PTx is associated with binding to carbohydrate on the target cell, an assay was developed to measure this binding activity of PTx to attempt to complement the ADP-ribosylation assay [130,131]. The ELISA based method initially looked at the ability of PTx to bind to plates coated with different glycoproteins or defined oligosaccharides. It was found that PTx bound preferentially to multiantennary *N*-glycans, especially fully sialylated structures such as fetuin. Pertussis toxoid lost the ability to bind to these structures [130]. It was found following validation that this biochemical assay system, comprising both the ADP-ribosylation test and carbohydrate binding assay, correlated with activity in the temperature HIST for 76 batches of seven different ACV products [132]. Subsequently, on behalf of WHO, an international collaborative study using three different commercially available types of ACV products showed that the biochemical assay system, was transferable between laboratories and was suitable for the three types of ACV products included in the study. The residual PTx activity of the three ACV products were different but had the same rank order in the in vitro assay system and the in vivo HIST [133]. This in vitro assay system was included as a potential alternative to HIST in “WHO Recommendations to Assure the Quality, Safety and Efficacy of Acellular Pertussis Vaccines” [92]. It could also be used to characterise PTd prepared by different chemical detoxification processes indicating its potential use for in-process control [134]. Nevertheless, the adoption of this assay system was limited due to two main criticisms: (a) this assay system does not measure all of the mechanisms required for PTx toxicity, such as translocation across the cell membrane, and (b) the assay system’s sensitivity has not been fully demonstrated using vaccines spiked with known concentrations of PTx as attempts to do so were not completely satisfactory for the ADP-ribosylation assay [135,136]. However, the carbohydrate-binding activity of PTx and different PTd products correlates with the rate of translocation into CHO cells using confocal microscopy with indirect immunofluorescence detection. This indicates that the carbohydrate binding activity could reflect the degree of translocation/internalization activity of PTx and PTd [77]. Furthermore, in a separate in vivo study using mouse footpad swelling model, carbohydrate binding activity in ACVs showed a positive relationship with the severity of local reactions after booster vaccination [137]. This again emphasises the important function of the B-oligomer and as an important determinant for PTx toxicity. As part of the EDQM BSP114 study the spiked vaccines included in the study to evaluate the CHO assays were also analysed by one participant in the biochemical assays. It was found that dose dependent results were observed for all spiked vaccines and that diluting the samples improved the overall results. The ADP-ribosylation activities of spiked vaccines was not statistically different from unspiked samples and this may be due to the high baseline ADP-ribosylation activity of some ACV products [112]. However, with appropriate assay criteria, both methods could be used to monitor product consistency during and after production [136].

### 2.5. Leukocytosis Promotion (LP) Test

Pertussis infection induces the production of large numbers of white blood cells (leukocytes) in humans, especially infants, and this is associated with poor clinical outcomes [138]. It was through studying the induction of leucocytosis in mice by WCV and bacterial culture supernatants that pertussis lymphocytosis-promoting factor (LPF) was first identified and purified. The LPF was subsequently identified and renamed as PTx [138,139,140,141]. The promotion of leukocytosis in the peripheral blood of mice can be used to determine any PTx activity in test samples by comparing the number of circulating leukocytes from mice injected with the test samples to those that receive control samples. The number of circulating leukocytes can be measured microscopically using a haemocytometer or an automated system such as a Coulter Counter [142]. The mean number of leukocytes three days after injection should not exceed 10 times the number before injection [143]. As with HIST there are many variable parameters associated with this test such as the route of administration (subcutaneous, intraperitoneal or intravenous), the number of days after injection in which the blood samples are (usually 3–6 days) and the number of mice per group. In a multi-laboratory study it was found that the LP test performed better than HIST and CHO in identifying three WCV products with high toxicity [142]. Although the LP test is included in the WHO Manual for the control of WCV combination vaccines it is not in the Recommendations to assure the quality, safety and potency of acellular pertussis vaccines [92,144]. There are several disadvantages associated with this method. Firstly, it is animal based and therefore there are ethical concerns about its use to measure PTx in vaccines. Like HIST the exact mechanism in which PTx causes the increase in white blood cells is unclear and there is a high degree of interlaboratory variability due to a lack of standardisation despite attempts to introduce harmonisation [138,142,145].

### 2.6. Other Cell-Based Assays

Since the ADP-ribosylation activity of PTx causes an increase in cellular cAMP in CHO cells along with clustering it may be possible to measure it to produce a more definitive quantitative estimate of PTx activity without the subjectivity of deciding when cells exhibit a clustering morphology. Hoonakker et al. have developed such an assay. Initially they used A10 cells and monitored the effect of PTx on the cells by measuring the intracellular levels of cAMP. This method could detect PTx activity, but it lacked sensitivity and robustness [146]. They improved the system by using novel reporter cell lines (CHO-CRE and A10-CRE) that stably express a reporter construct that is responsive to cAMP levels. Both cell lines can detect PTx in a concentration-dependent manner but only the CHO-CRE cell line can detect PTx in a multivalent vaccine matrix at levels similar to HIST [147]. The sensitivity of this method has been reported as 0.2 IU/mL for pure PTx [112]. The CHO-CRE (CHO-cAMP-PTx reporter cell assay) based method was used in the collaborative study for the establishment of the WHO 2nd IS for PTx and the activity of the new standard relative to the 1st IS was comparable to the results observed by participants that performed the standardised CHO cell clustering assay [103]. This indicates that it produces results similar to the CHO clustering assay and therefore has the potential to be a less subjective cell-based assay, but vaccine adjuvant associated cytotoxicity may also present a problem.

The cell clustering and changes in cAMP levels observed in the cell-based assays discussed previously are associated with the ADP-ribosylation activity of PTx following its binding to the cells and subsequent internalisation. While the ADP-ribosylation pathway is clinically important it may not be the only physiological effect of PTx and the B-subunit has been shown to induce an ADP-ribosylation independent pathway on its own. However, the two pathways differ in that ADP-ribosylation occurs more rapidly and at much lower concentrations of PTx than the ADP-ribosylation independent pathway [148]. Therefore, several studies have used gene expression profiling of human cell lines and animals exposed to PTx by DNA microarray as a way to determine all aspects of PTx activity. Through these methods a number of possible biomarkers for toxin activity have been identified [143,148,149,150]. These studies may further improve the understanding of PTx toxicity and lead to more accurate assays.

## 3. Discussion and Conclusions

Due to recent outbreaks of pertussis infection, there is a continued interest in, and an urgent need for, the development of new and improved ACV vaccines. Ideally these will induce longer lasting, more cell-based immunity as well as reducing transmission of infection to offer the potential for herd protection. There may also be WCV products developed that have reduced toxicity. Therefore, methods to measure PTx activity continue to be required to support the development and production of new vaccines.

There are many challenges in measuring residual PTx activity in ACVs. These include the diversity of vaccine preparations, differences in pertussis antigen composition and detoxification methods. Also, various purification processes and formulations are used. The basis of the current regulatory approaches for testing residual activity is to show consistency with clinical lots or equivalents. However, a precise quantitative assay would be preferable to a limit test. For the characterisation of new products and formulations or for performing pre-clinical evaluation, any assay method that is validated and optimised for use should use a stable reference toxin calibrated in terms of bioactivity (IU) to allow calculation of relative activity. Inclusion of such a reference toxin will help to define assay sensitivity and to express results in IU with traceability to IS. Assay sensitivity can be established with spiking experiments to show that the assay can detect a defined amount of spiking dose (e.g., 2IU) and the activity limits for this spiking dose should be defined during validation stage. Routine assays should include the spiking dose and the activity should fall within the pre-defined limits and included as one of the validity criteria. The chosen assay should establish limits for the ‘safe’ amount of PTx activity in IU based on a panel of products with a known history of safety. Assays should define an upper limit for residual active PTx. Manufacturers will need to prove that the chosen method is effective to measure residual PTx activity in their product as optimisation maybe required for their specific products. Ideally any assay for measuring PTx activity will be in vitro based and have the same sensitivity as HIST. This would reduce the number of animals used in the production of ACVs.

There are a number of stable PTx references available from the WHO (NIBSC code: 15/126) and EDQM (Catalogue No. Y0000021) which have been calibrated in IU for HIST (lethal method) and the standardised CHO cell-clustering assay.

All the methods described in this review can detect levels of PTx activity associated with safe vaccine products. Once optimised and validated, they, all have the potential to assess residual and reversion to toxicity. Assays may also require vaccine specific modifications for final product testing due to the potential interference by adjuvants or excipients. These modifications may include diluting the vaccines to overcome cytotoxic adjuvant, testing only vaccine pellets as opposed to the complete formulation and/or desorbing antigens from adjuvants. Specificity and detection limits are very important for any methods to be considered suitable for measuring PTx activity in ACVs. Due to the disadvantages and ethical concerns associated with HIST, and animal use in general, it is important that alternative methods used to measure PTx activity should be based on in vitro technology. All the methods mentioned here have their advantages and disadvantages. The ideal in vitro assay would measure all the steps in PTx toxicity (carbohydrate binding, translocation and ADP-ribosylation activity) and be suitable for the final formulation of the vaccine in the presence of adjuvants. Due to the high degree of variability and methods of HIST it may not be possible to find a correlation between it and an in vitro alternative. The question of validation of alternatives to HIST have been discussed elsewhere [93].

The recent decision to remove the requirement for testing for residual and reversion to toxicity in final formulated vaccines from the European Pharmacopoeia once the detoxification process has been demonstrated to be stable means that not only does HIST not have to be performed on final formulations but none of the in vitro methods considered as alternatives to HIST are required [106]. The activity of purified PTx and later the PTd will be determined upstream by the manufacturer using a CHO cell clustering-based assay. This will lead to a reduction in the use of animals in countries and for well-established products where testing adheres to the European Pharmacopoeia guidance. Tests for residual toxicity and reversion to toxicity will still be required for new products, ideally these will be validated in vitro assays, but in vivo tests may be necessary.

Furthermore, considering the fact that the majority of ACV products currently used globally are long established, it may be possible for other regulatory authorities to follow the example of the EDQM and remove the requirements for testing for residual and reversion to toxicity from final products once they are satisfied that the detoxification is robust. While this too would reduce the numbers of mice used in testing there always remains the possibility to use validated in vitro methods to monitor consistency of final products if evidence suggests that it is required.

## Figures and Tables

**Table 1:** Histamine sensitisation test requirements specified in regulatory guidelines (taken from Arcinega et al., 2016 and Hoonakker et al., 2017 [93,102]).

Requirement	WHO	EU ^1^	USA	Canada	China	Japan
Requirement according to	TRS 979	Ph. Eur.	License Dossier	Ph. Eur.	Ph. Chin.	Ph. Ja.
Final lot (or bulk)/in process	Final bulk (one or more Dilutions)	In process and/or final lot	Final lot (or bulk)	Final lot	Bulk and final bulk	Final lot
Injected vaccine volume	1 or 2 HD	2 HD	0.5 mL	2 HD	0.5 mL	0.5 mL
Vaccine sample storage	1 or more dilutions, 4 °C and or 37 °C	4 °C and 37 °C	4 °C	4 °C	4 °C and 37 °C	4 °C and 37 °C
Negative Control	Diluent or none	Diluent	Diluent	Diluent	n.s.	n.s.
Positive control (and number of dilutions)	PTx (one or more dilutions)	PTx (one dilution)	PTx (one)	PTx (one dilution)	PTx (several dilutions)	PTx (several dilutions)
Number of mice per group	Ten or an appropriate number	5	20	5	10	10
Minimal number of groups	App. 5	3	App. 3	2	App. 5	App. 6
Histamine challenge	defined dose of histamine (usually 1 or 2 mg)	2 mg of histamine	1 mg of histamine	2 mg of histamine	2–4 mg of histamine	4 mg of histamine
Time interval between sample administration and challenge	4–5 days	5 days	5 days	5 days	4 days	4 days
Observation period	30 min–24 h	24 h	24 h	24 h	30 min	30 min
Minimum animal number 1 test	App. 50 animals	15 animals	App. 60 animals	10 animals	App. 50 animals	App. 60 animals
Readout parameter	Temperature decrease or death	Death	Death	Death	Temperature decrease	Temperature decrease
Acceptance criteria	Residual activity of PTx or the number of animals that die is not higher than specified by the NRA. If a vaccine lot fails in a single test, it should pass 2 additional test for release.	The vaccine complies if in the group that receives the vaccine stored at 2–8 °C or 37 °C, there are no deaths or no more deaths than in the group that receives the reference vaccine. If one mouse dies in one or both of the vaccine groups, repetition is allowed with the same number of mice or more. The vaccine is accepted when overall death rate is 5% or less.	One undiluted single human dose of 0.5 mL sensitizes no more than 10% of mice injected. If the vaccine fails to meet the criterion in a first test, it should pass 2 additional tests.	The vaccine complies with the test if in the group that receives the vaccine, there is no more than one death. If more than one mouse dies in the negative control group or the vaccine group, repetition is allowed with the same number of mice or more. The vaccine is accepted when overall death rate is 6.25% or less.	The histamine-sensitizing toxicity of both test samples at 4 °C and 37 °C shall be no higher than 0.8 HSU/mL in mice upon statistical analysis.	The histamine-sensitizing toxicity of both test samples at 4 °C and 37 °C shall be no higher than 0.4 HSU/mL in mice upon statistical analysis.
Validity criteria	1. Less than 5% deaths in the negative control group. 2. Demonstrated sensitivity of mice strain. 3. When linearity of log dose-response to PTx is demonstrated 1 positive control group suffice.	1. No mice die in the negative control group. 2. Sensitivity of the mice is demonstrated (e.g., 30% of the mice die in the positive control group). 3. A suitable mouse strain has a toxin LD50 between 6 IU and 50 IU.	1. PTx control group should show that mice used are sensitized by a dose of PTx below 100 ng, in terms of the HSD50. 2. No more than 10% of mice should die in the negative/diluent group.	1. There are at least 16 mice challenged per group. 2. No more than one mouse dies in the negative control group. 3. Sensitivity of the mice is demonstrated, i.e., at least seven mice die in the positive control group (=43.75%, mice injected with 400 ng of PTx).	n.s.	n.s.

^1^ The requirement for testing for residual and reversion to toxicity will be removed from Ph. Eur. [106]. App.: approximately; HD: (human dose); n.s.: (not specified); Ph. Chin.: Chinese Pharmacopoeia; Ph. Eur.: European Pharmacopoeia; Ph. Jap.: Japanese Pharmacopoeia; PTx: pertussis toxin; TRS: Technical Report Series; WHO: World Health Organization.
